# Numerical Analysis on Dynamic Response of CFRP-Wrapped RC Columns under Lateral Impact Loading

**DOI:** 10.3390/ma16062425

**Published:** 2023-03-18

**Authors:** Tao Liu, Xiaoqing Xu, Lin Chen, Sanghee Kim, Seongwon Hong

**Affiliations:** 1 School of Civil Engineering, Hunan University of Science and Technology, Xiangtan 411201, China; 2 Department of Architecture Engineering, Kyonggi University, Suwon 16227, Republic of Korea; 3 Department of Safety Engineering, Korea National University of Transportation, Chungbuk 27469, Republic of Korea

**Keywords:** CFRP, RC columns, lateral impact loading, finite element analysis, LS-DYNA, parametric analysis, impact velocity, axial compression ratio, damage mode, impact resistance

## Abstract

This paper presents a numerical study examining the dynamic response and resistance mechanism of reinforced concrete (RC) columns strengthened with or without carbon-fiber-reinforced polymer (CFRP) wraps under lateral impact loading by using the software LS-DYNA. First, the information of eight column models was briefly introduced as part of the laboratory experimental program from the literature. Secondly, finite element (FE) models were established in terms of the geometries of impact tests. Then, a detailed comparison between numerical results and experimental results was made, and FE models showed a relatively high simulation accuracy. Subsequently, a series of parametric analyses were carried out with a focus on the effects of axial compression ratio, the boundary condition at the column top, the layer number of CFRP wraps, and the impact velocity and impact height on the dynamic responses of plain and strengthened columns. The results demonstrated that the CFRP retrofit mechanism was not activated during the initial Stage-I when the impact force rapidly increased to the first peak and then decreased to zero. CFRP strengthening came into play in the second stage, Stage-II, and affected the response of the shear force and moment along the column height, as well as had a great influence on the control of shear damage. The dynamic response of RC columns was more sensitive to the impact velocity than to other parameters, regardless of whether CFRP wrapping was applied. The axial compression ratio would have a different influence on the column failure mode if the impact velocity was varied. The variation in impact height and boundary condition at the column top had little influence on the damage mode of strengthened columns.

## 1. Introduction

Fiber-reinforced polymer (FRP) composites have been widely employed in the field of retrofitting concrete structures to resist static, fatigue, and seismic loads due to the advantages of light weight, high strength, excellent corrosion resistance, and easy installment. Strengthening concrete columns by full FRP wraps can form a hoop effect to improve the axial and transverse load-bearing capacities, enhance the lateral stiffness, and restrict the lateral deformation [1,2,3]. In recent years, the possibility of existing reinforced concrete (RC) pier columns suffering lateral impact loads is gradually increasing because of the frequent occurrence of vehicle and ship collisions with RC pier columns. When subjected to lateral impact loading near the ground, RC pier columns are prone to suffer shear damage or failure. It is a potential approach of applying full FRP wraps to strengthen RC pier columns in shear to improve the resistance against lateral impacts.

Some researchers [4,5,6,7,8,9,10,11,12,13,14] found that strengthening with carbon-fiber-reinforced polymer (CFRP) composite wraps could improve the shear impact resistance of RC beams, prevent the development and expansion of shear cracks, and eventually restrict the shear damage. Suter et al. [15] found earlier that wrapping RC columns transversely with aromatic fiber (Kevlar) composites could significantly enhance the shear impact resistance of columns. Ferrier et al. [16] conducted lateral impact tests on RC columns strengthened with CFRP wraps and found that using CFRP could reduce the lateral deformation of columns and increase the impact resistance by about 88%. Isaac et al. [17] stated that CFRP enhancement could reduce the deformation and control the shear damage degree of RC columns under impact. Gurbuz et al. [18] investigated the effect of aromatic-fiber-reinforced polymer (AFRP) wraps on the deformation and shear impact resistance of RC columns under impact, and demonstrated the change in failure mode from shear to flexure. Sha et al. [19] found that increasing the layer number and tensile strength of CFRP wraps could improve the lateral impact resistance of RC columns. Dong [20] and Ding [21] reported that the enhancement with FRP could increase the lateral stiffness of RC columns, reduce the deformation, and change the failure mode from shear to flexure. Wang et al. [22] stated that the initial kinetic energy level of the impactor had a significant influence on the impact behavior and damage degree of CFRP-strengthened RC columns. Xu et al. [23] tested RC circular cantilever columns strengthened in shear with CFRP wraps by horizontally lateral impacts and showed the results that a sufficient amount of CFRP wraps based on the static design requirement of shear retrofitting could effectively control the shear damage and change the failure from shear to flexure. Zhou et al. [24] found that CFRP strengthening according to the seismic reinforcement requirements could reduce the vulnerability of RC pier columns under vehicle collision. Liu et al. [25,26] completed the asymmetric transverse impact tests of CFRP-wrapped RC columns and found that transverse CFRP wrapping could reduce shear damage but increase the suddenness of failure, and the wrapping scheme and layer number had little influence on the peak impact force. Liu et al. [27] numerically analyzed the effects of the type, layer number, and fiber orientation of FRP wraps on the dynamic responses of RC pier columns under vehicle collision, and accordingly proposed two dimensionless damage indexes to justify the shear failure. Li et al. [28,29] found that external FRP wrapping could effectively enhance the vehicular impact-resistance of bridge piers, and, to some extent, avoid bridge collapses under collisions at high speed.

Up to now, the research on the dynamic response and impact resistance of FRP-strengthened RC columns under lateral impact is still limited. The influence of some critical factors such as the axial compression ratio of RC columns, boundary condition, FRP properties, impact velocity, and impactor mass is still unclear. In recent decades, the finite element analysis method has been rapidly developed and extensively applied in various fields [30,31]. Hence, this paper attempts to conduct finite element analysis on the effects of those factors by using commercial software LS-DYNA 971 [32].

First, a brief introduction of an impact test program from the literature [21,23] was finished. Secondly, three-dimensional finite element (FE) models were established based on the impact test, and then a detailed comparison between numerical results and experimental results was conducted in order to validate the FE models. Thirdly, the validated FE models were used to perform detailed parametric analyses to deeply study the effects of axial compression ratio, boundary condition, the layer number of CFRP wraps, and impact velocity and height on the dynamic response and failure mechanism of RC columns under lateral impact loading. Finally, the variation in shear force and moment as well as CFRP stress was analyzed to understand the contribution mechanism of CFRP wraps to the impact resistance of columns.

## 2. Materials and Methods—Brief Introduction of Experimental Program

**Figure 1:**
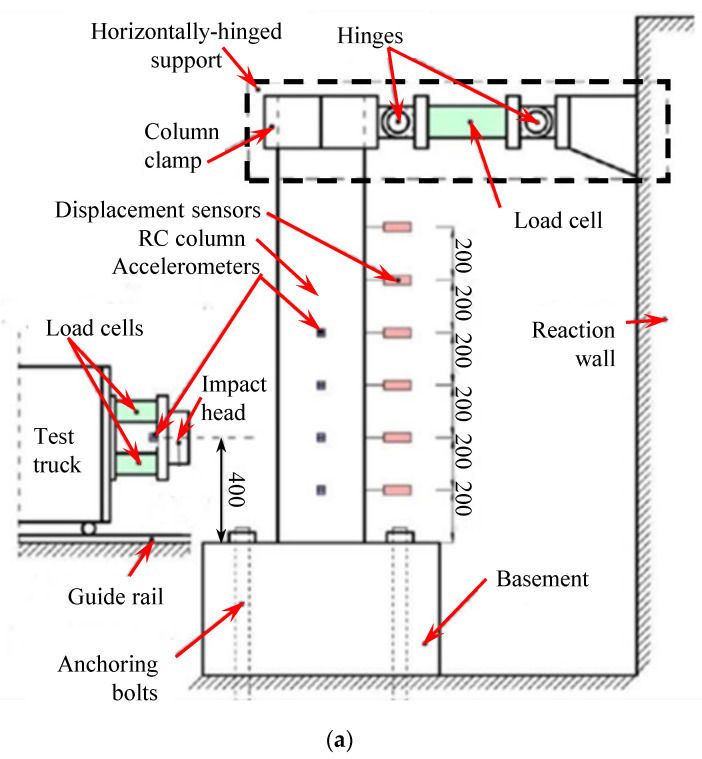

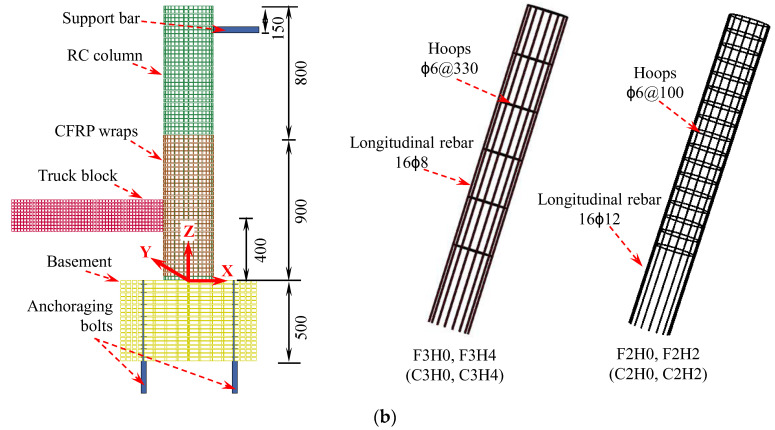
Numerical modeling of a typical RC fixed-hinged column strengthened with CFRP: (**a**) schematic diagram of impact test setup [21]; (**b**) FE model (Note: Units are in mm).

The literature [21,23,33] reported a series of lateral impact tests on RC columns wrapped with CFRP. Eight typical column specimens labeled C2H0, C2H2, C3H0, C3H4, F2H0, F2H2, F3H0, and F3H4 were selected as the basis to build FE models. All eight specimens had an identical circular section with a diameter of 330 mm. As shown in Figure 1, the height of each column was 1700 mm above the basement, which was fixed to the ground by four anchoring steel bolts. The bottom of all the columns could be considered as being fixedly constrained. The tops of columns C2H0, C2H2, C3H0, and C3H4 were freed, which formed a cantilever boundary, and these columns were labeled as cantilever columns, while specimens F2H0, F2H2, F3H0, and F3H4 were constrained by a horizontal hinge support system at the top and were considered as fixed-hinged columns, as presented in Figure 1a. Sixteen longitudinal rebars were evenly distributed around the circumference of each column with a concrete cover of 25 mm thickness. The covering height of CFRP wraps was 600 mm and 900 mm from the top surface of the basement for cantilever columns and fixed-hinged columns, respectively. Other details are summarized in Table 1, including the average cylinder compressive concrete strength for each column. The test truck having a total mass of 1582 kg impacted the columns at a velocity of 4.5 m/s. The center height of the frontal impact surface of the truck was 400 mm from the top surface of the basement. Table 2 provides the material parameters of steel rebars used in tests. Unidirectional carbon fiber sheets with a nominal thickness of 0.167 mm for one layer were utilized by having a tensile strength of 3494 MPa and elastic modulus of 240 GPa. The matrix of carbon fibers adopted the epoxy resin adhesive having a tensile strength of 40 MPa, elastic modulus of 4 Gpa, compressive strength of 70 MPa, and shear strength of 20 MPa. Other details of the tests can be referred from the literature [21,23,33].

## 3. Validation of FE Model

### 3.1. FE Modeling

The paper employed LS-DYNA software to establish three-dimensional FE models of CFRP-wrapped RC columns based on the tests. The details of how to build the models of cantilever columns C2H0, C2H2, C3H0, and C3H4 can be found in the literature [27], while the fixed-hinged columns F2H0, F2H2, F3H0, and F3H4 had the same modeling method except for a horizontal hinge support. It was assumed that the test truck was simply modeled by a solid block having the same-size impact surface, impact velocity, and mass. The top horizontal hinge support system was simplified to a horizontal support bar. One end point of this bar was constrained by all the displacement but allowed for all the rotation, while another was connected to the column top point corresponding to the center of the horizontal hinge support system. An eight-node hexahedron solid element with a single integration point was used to model the column concrete, basement, and the simplified truck block. A two-node beam element with a 2 × 2 Gauss quadrature integration and a Hughes–Liu element formulation were employed for the longitudinal steel rebars and transverse hoops, respectively. The anchoring steel bolts were modeled using a truss element. The CFRP wraps were simulated by using a four-node quadrilateral thin shell element with a Belytschko–Tsay element formulation. A spring discrete beam element was used for the horizontal support bar at the column top. The mesh size was determined as about 25 mm for the column concrete, rebar, CFRP sheets, and simplified truck block and as about 50 mm for the basement, based on a mesh convergence study to strike a balance between computational cost and reasonable results. The bonds between concrete and rebar and between concrete and CFRP were assumed to be perfect by sharing their nodes together. The material model Mat_072R3 was used to characterize the dynamic behavior of concrete, considering the strain rate by inputting the CEB model modified by Malvar and Crawford [34]. The deletion of concrete elements was simulated by defining an erosion algorithm with a maximum principal strain value of 0.3 beyond which the concrete elements would be deleted. A stiffness-based hourglass mode was defined for concrete elements to avoid the zero-energy deformation mode that had an influence on the solution results. A piecewise linear-plastic material model Mat_024 with a viscoplastic formulation was used for all steel rebars. The fracture of rebars could be simulated by the element deletion, which would occur when the strain exceeded the input maximum plastic strain. The strain rate effect was taken into consideration by using the Cowper–Symonds model [35] to scale the tensile stress. CFRP wraps were modeled by using an enhanced composite damage material model Mat_054. This model contained four types of stress-related failure criteria, such as tensile fiber rupture, compressive fiber buckling, tensile matrix cracking, and compressive matrix failure. For tensile fiber rupture, an additional strain-based criterion could be defined by inputting a maximum tensile strain beyond which the fiber element would be deleted. Zhang et al. [36] found that the strain rate had little influence on the tensile strength and elastic modulus of FRP when being less than 10 s^−1^. Accordingly, the strain rate effect on CFRP was not considered in this analysis herein. A spring discrete material MAT_196 was used for the horizontal support bar. The axial compressive and tensile stiffnesses of the discrete beam were determined to be 40 kN/mm and 10 kN/mm, respectively, by conducting trial and error analysis, which could simulate well the lateral constraint effect of support in the tests. For the simplified truck block, column basement, and steel anchoring bolts, an elastic material model Mat_001 was employed. A penalty-based contact algorithm CONTACT_Automatic_Surface_To_Surface was defined between the truck and column or CFRP. All the parameters were defined with default values, except for the static and dynamic friction coefficient of 0.3.

### 3.2. Model Validation

**Figure 2:**
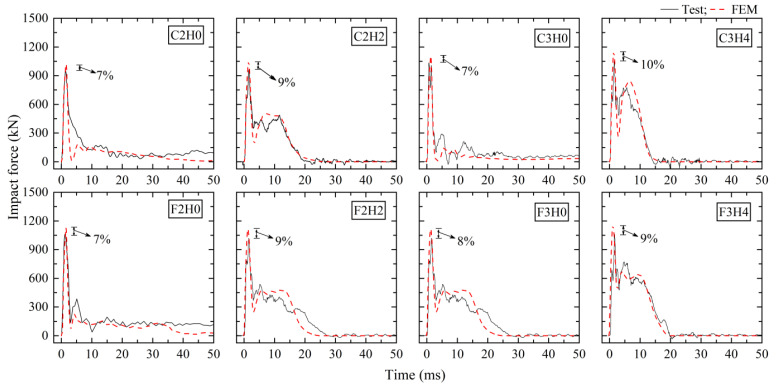
Comparison between numerical results and test results of impact force.

**Figure 3:**
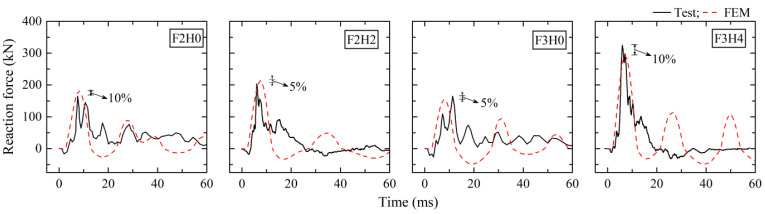
Comparison of reaction force of fixed-hinged columns.

Figure 2 describes the comparison between numerical and experimental data of impact loads. FE models showed a good resemblance of impact force time histories to experimental results. The differences in peak impact force were less than 10%. Figure 3 shows the comparison of reaction force time histories of fixed-hinged columns F2H0, F2H2, F3H0, and F3H4. Note that the axial force of the horizontal support bar was output from the FE model as the reaction force. It was observed that the main impulses of reaction force in the initial stage was able to be simulated well by FE models with an error of peak reaction force less than 10%, but there was an obvious deviation after the peak. The reason might be that there was a connection gap in the horizontal hinge support system in the tests, and this had little influence on the reaction force during the action of the main impulse but a great influence on the horizontal stiffness in the late stage of free oscillation.

**Figure 4:**
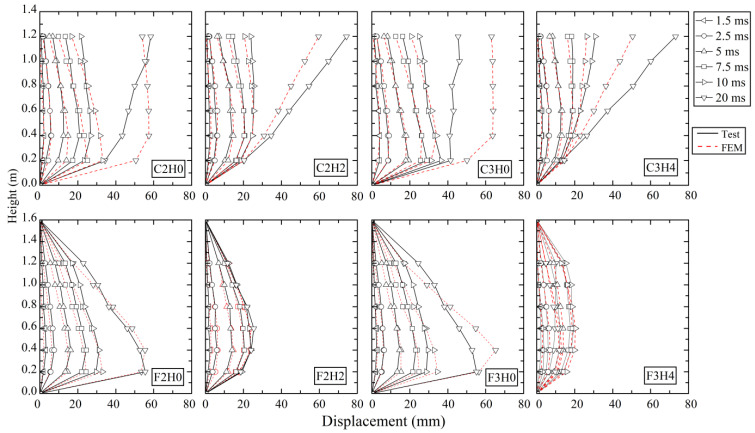
Comparison of horizontal displacement distribution.

In Figure 4, numerical results of the horizontal displacement distribution along the height of the column agreed well with the experimental results, especially for the fixed-hinged columns, though there were some errors at the upper part of cantilever columns. This might be because the failure locations of cantilever column models were slightly different from the tests, leading to a different horizontal inertial force and consequently different displacement at the upper part after failure.

**Figure 5:**
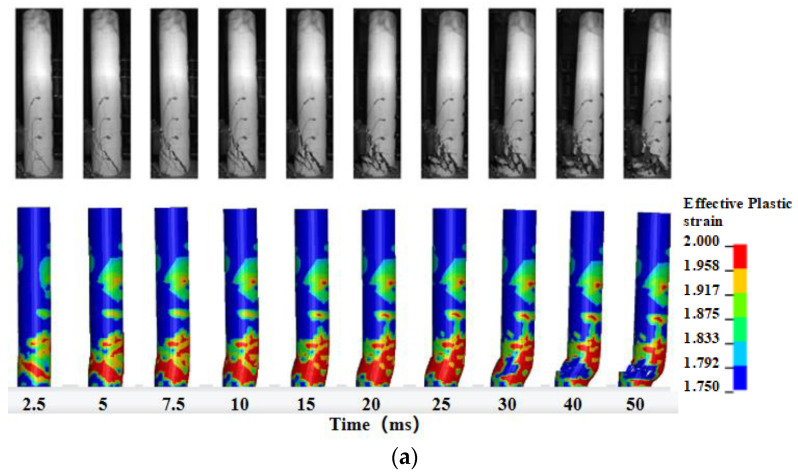

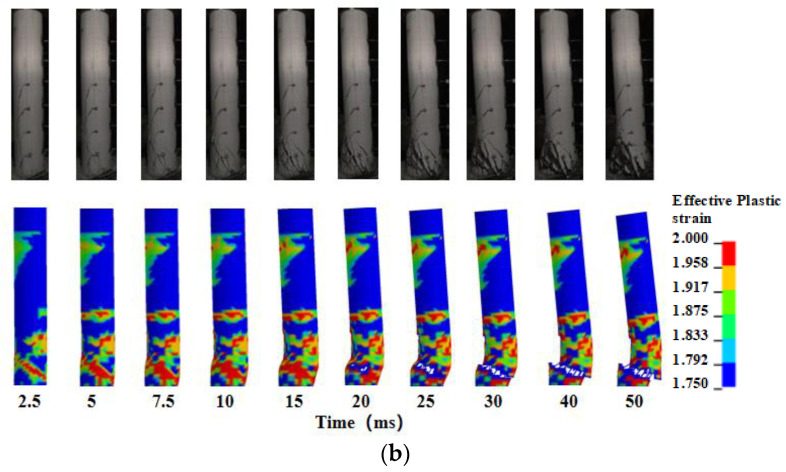
Comparison of impact process: (**a**) column C3H0; (**b**) column F3H0.

**Figure 6:**
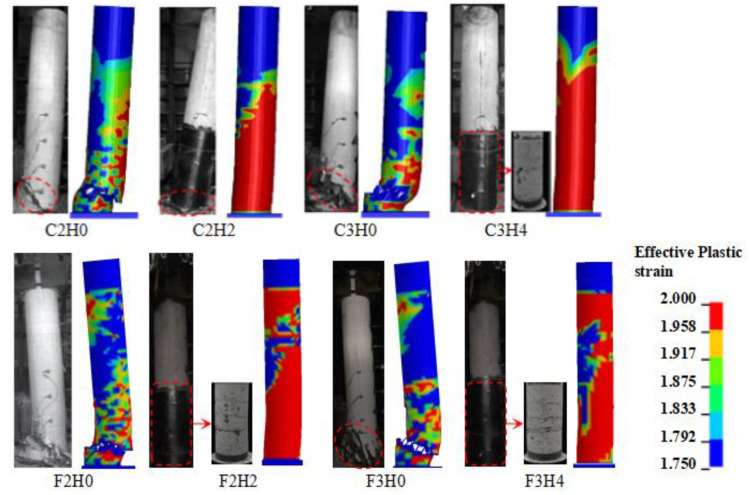
Comparison of final failure modes.

Figure 5 demonstrates the comparison of the impact damage evolution process of specimens C3H0 and F3H0. The FE models could simulate well the occurrence and development of critical shear cracks, shear failure, and flexural damage on the back of the impact zone of columns, though there were slight deviations in the inclination angle of critical shear cracks for both columns. The final failure modes of eight columns are compared between numerical results and experimental results in Figure 6. The shear failure mode was dominant in the unstrengthened columns, and captured well by the FE models. CFRP-wrapped columns presented a flexure-dominant failure mode, while the FE models could simulate the main damage pattern but show a more severe strain contour. On the basis of these comparisons between the numerical results and experimental results mentioned above, the developed FE models could be considered being capable of predicting well the dynamic response of plain and CFRP-wrapped RC columns under lateral impact loading.

## 4. Parametric Analysis

Based on the validated FE models, a detailed parametric analysis was carried out in this section. The unstrengthened RC column having the same reinforcement details as C3 and F3 group columns served as the basis for the parametric analysis. There were sixteen 12 mm diameter longitudinal steel rebars and 6.5 mm diameter hoops at a spacing of 330 mm. The cylinder compressive strength of concrete was 30 MPa. If subjected to lateral static concentrated loading at a height of 400 mm from the basement, this unstrengthened cantilever RC column would have an insufficient shear capacity and suffer shear failure according to the theoretical calculation in the literature [23].

The parametric values are listed in Table 3, including the boundary condition at the top of the column, the axial compressive ratio, the layer number of CFRP wraps, the impact height of the truck block from the top surface of the basement, the impact velocity, and the impact mass. The label of each impact case was defined as follows: the first letter of C or F means the cantilever (C-series) or fixed-hinged (F-series) boundary conditions, respectively; the second letter of L, M, and H means the impact height of 400 mm, 600 mm, and 800 mm, respectively; the following number stands for the value of 10 multiplied by the axial compressive ratio; the rear combination “FX” indicates the use of FRP wraps with the layer number of X; the last combination “Vx” indicates the impact at a velocity of x m/s. For example, the case “CL1F2V2” indicates that a C-series (cantilever) column wrapped with a two-layer CFRP having an axial compressive ratio of 0.1 was laterally impacted at a velocity of 2 m/s with a lower impact height of 400 mm from the basement.

It is worth noting that the value of the axial compressive ratio was determined based on the theoretical value of axial bearing capacity of unstrengthened RC columns, and the influence of CFRP wraps was not considered. For the impact cases having an axial compression ratio, the analysis process was divided into two phases. The first phase in the initial period of 0.02 s was the application of axial loading, which was posed on all nodes of the column top surface and kept constant for subsequent impact scenarios. Column vibration might be induced by the rapid application of axial loading and, hence, mass damping was defined to make the column stabilize quicker. The second phase was the impact of the truck model on the column. In order to facilitate comparison with the impact case without axial loading, the time point of 0.02 s was taken as the new origin of the time axis for the case having an axial compression ratio.

**Figure 7:**
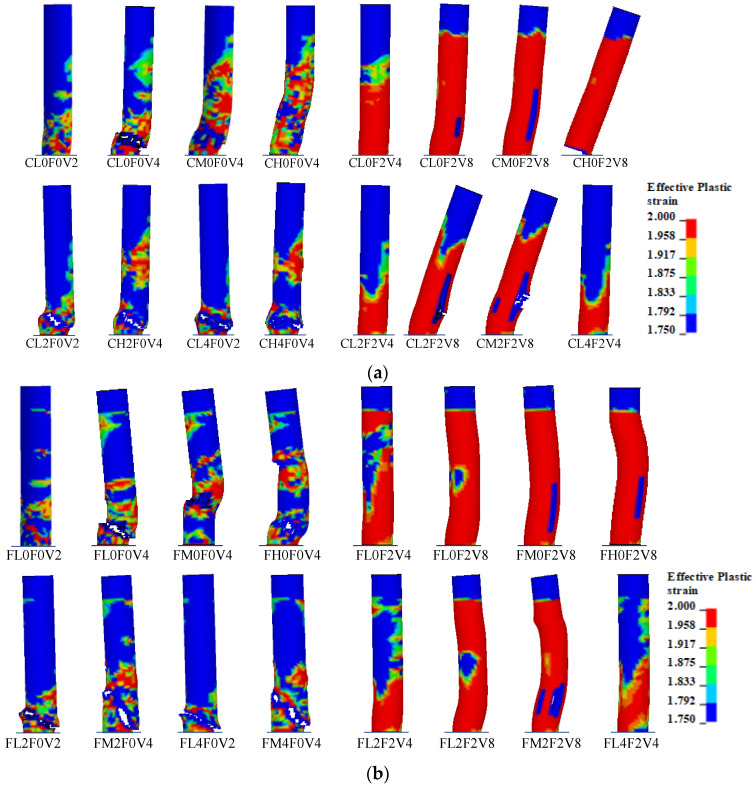
Failure modes: (**a**) C-series columns; (**b**) F-series columns.

As shown in Figure 7a, for C-series cases without the axial compressive ratio of *μ*, plain columns that were designed to fail in the shear mode under static loading developed obvious shear damage at the impact velocity *v* of 2 m/s for case CL0F0V2, and suffered more severe shear failure when *v* increased to 4 m/s for case CL0F0V4. Strengthening with two-layer CFRP wraps enhanced the column of case CL0F2V4 to sustain the impact with a higher velocity, and the flexure-shear-combined damage mode became more serious with the increase in *v* to 8 m/s for case CL0F2V8, which was considered as the critical impact velocity *v*_cr_ leading to severe failure. Increasing the CFRP amount to four layers showed a similar damage mode, which is not herein shown, but increased the value of *v*_cr_ to 10 m/s. Accordingly, CFRP wrapping could improve the impact shear resistance of RC columns, control shear damage, and change the failure mode from shear to flexure-shear or even to flexure. With the increase in impact height *h* from 400 mm to 800 mm, the damage mode was still dominant in the shear pattern for plain columns CL0F0V4, CM0F0V4, and CH0F0V4, while it tended to the flexure-dominant pattern for strengthened columns CL0F2V8, CM0F2V8, and CH0F2V8. Because the plain column was still static-shear-deficient when the lateral load was posed on the height of 800 mm, the damage mode was unchanged, while columns strengthened with CFRP wraps obtained an improvement of shear resistance and exhibited a flexure-shear mode. The increase in impact height would speed up the change to the flexure mode due to the demand increase of the moment at the column bottom. Therefore, the variation in impact height would have no obvious influence on the main damage mode. The increase in *μ* to 0.2 caused a more concentrated and severe shear damage to the plain column at the impact velocity *v* of 2 m/s for case CL2F0V2, and a further increase in *μ* to 0.4 would aggregate the damage for case CL4F0V2. However, when *v* of 4 m/s was smaller than *v*_cr_ of 8 m/s, strengthened columns with two-layer CFRP wraps would have a reduction in damage degree with the increase in *μ* from 0 to 0.4, by comparing case CL0F2V4, CL2F2V4, and CL4F2V4. This indicates that when the impact with a lower velocity was insufficient to cause shear damage, the application of axial compression ratio would be beneficial to enhance the impact resistance of the column. As the impact velocity reached *v*_cr_ of 8 m/s, the increase in *μ* to 0.2 caused severe damage for case CL2F2V8. This implied that the effect of axial compression ratio on column damage would depend on the magnitude of impact velocity. The effect of *μ* on the damage to columns impacted at a higher impact height *h* was similar to that at lower *h*. From Figure 7b, the variation in boundary condition at the column top to the hinged support showed a similar effect of axial compression ratio, the layer number of CFRP wraps, impact velocity, and impact height on the column damage. Especially for the cases under the lower impact height of 400 mm, the hinged constraint at the top had almost the same influence on the column damage to the cantilever condition.

### 4.1. Response of Impact Force

**Figure 8:**
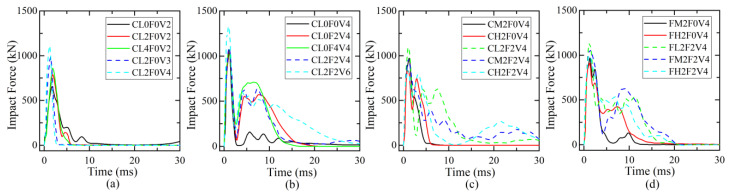
Parametric effects on time histories of impact force: (**a**) impact velocity and axial compression ratio; (**b**) number of CFRP layers and axial compression ratio; (**c**) impact height and number of CFRP layers; (**d**) impact height and number of CFRP layers.

The effect of different parameters on the time history curves of impact force is illustrated in Figure 8. The response curve of the impact force was generally featured by two stages: Stage-I was the initial stage when the impact force rose rapidly to the first peak and then dropped sharply to close to zero, forming a triangular main pulse; Stage-II was the following stage when the impact force experienced a second increase to the second peak smaller than the first peak, a long time duration of free vibration, and finally a decrease to zero. As shown in Figure 8a, the increase in impact velocity from 2 m/s to 4 m/s and axial compression ratio *μ* from 0 to 0.4 produced almost similar curve shapes of impact force, with an increase in the first peak and a slight decrease in Stage-I time duration. Due to suffering severe shear failure in Stage-I, plain C-series columns did not have an obvious Stage-II response. From Figure 8b, under the same impact velocity, the increase in CFRP layer number from 0 to 4 had almost no influence on the Stage-I response of the impact force, but a significant influence on that of Stage-II. Strengthening with sufficient CFRP wraps could improve the impact shear resistance to prevent shear failure in the Stage-I response and help to develop the Stage-II response of impact force. The higher the number of CFRP wraps, the higher the second peak of impact force. Compared with column CL0F2V4, the increase in *μ* to 0.2 for column CL2F2V4 showed a similar impact-force curve but with little increase in the second peak, while the increase in impact velocity for column CL2F2V6 significantly increased the first peak and Stage-II time duration but with a similar Stage-I time duration and second peak. The effect of impact height *h* on the impact force histories for C-series columns is illustrated in Figure 8c. As *h* increased from 400 mm to 800 mm, the impact force of plain and strengthened columns tended to experience a slight drop in the first peak and a rise in the second peak, as well as a longer time duration. For F-series columns, as shown in Figure 8d, the increase in impact height showed an almost similar effect on the impact-force response. From the comparison between Figure 8c,d, the boundary condition at the top of the column had less influence on the impact force for columns impacted at a lower height of 400 mm. When *h* was 600 m or 800 mm, the impact force presented a close first peak, but different curve shapes for C-series and F-series columns, especially in Stage-II. This indicates that the boundary condition at the top would have a significant influence on the Stage-II response of the impact force. From Figure 8, the impact force in Stage-I was more sensitive to the impact velocity than to other parameters, while the CFRP layer number, impact height, and boundary condition at the top had an obvious influence on the Stage-II response.

### 4.2. Response of Lateral Displacement at the Impact Height

**Figure 9:**
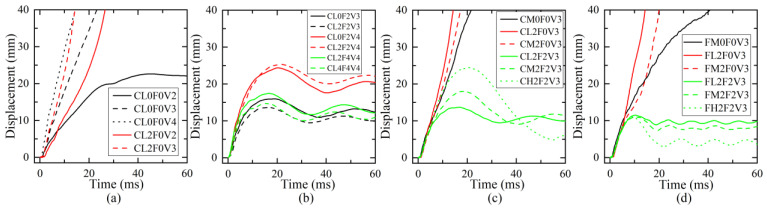
Parametric effects on time histories of lateral displacement: (**a**) impact velocity and axial compression ratio; (**b**) number of CFRP layers and axial compression ratio; (**c**) impact height and number of CFRP layers; (**d**) impact height and number of CFRP layers.

Figure 9 gives some time histories of lateral displacement at the impact height to investigate the parametric effect. There were two typical curve shapes of displacement history. One presented an almost linearly increasing pattern, and the other underwent a parabolic-pattern growth and then free oscillation after the peak to the final residual displacement. It is worth noting that during the Stage-I response of impact force with a duration of about 2.5 ms, the displacement developed slightly but significantly during the Stage-II response. After the impact force decreased to zero, the displacement entered into the free oscillation stage.

It can be seen from Figure 9 that the lateral displacement was more sensitive to the impact velocity than other parameters and increased significantly with the increase in impact velocity. The axial compression ratio *μ* had a different influence on lateral displacement for plain and strengthened columns, which had a coupling effect with the impact velocity. If the impact had sufficient velocity to cause serious shear damage, the increase in *μ* would result in a significant increase in lateral displacement, evidenced by plain columns as shown in Figure 9a. However, if the impact velocity was too small to induce obvious shear damage, the increase in *μ* would help to reduce the lateral displacement, because the axial force on the column could enhance the lateral shear capacity to some extent. This was proved in Figure 9b by the comparison between columns CL0F2V3 and CL2F2V3, and between columns CL2F4V4 and CL4F4V4. Therefore, the coupling effect of critical impact velocity and axial compression ratio needed to be considered. In addition, the increase in CFRP amount could provide a stronger transverse confining effect to enhance the axial load capacity of the column to achieve a higher critical value of *μ*. From Figure 9c,d, the increase in impact height apparently increased the lateral displacement for C-series strengthened columns, while it slightly decreased the displacement for F-series strengthened columns. The variation in impact height would change the vibration frequency of displacement after the peak. The change in boundary condition at the top of the column from the cantilever to the hinge reduced the displacement under the same impact height and velocity.

### 4.3. Distribution Curve of Lateral Displacement

**Figure 10:**
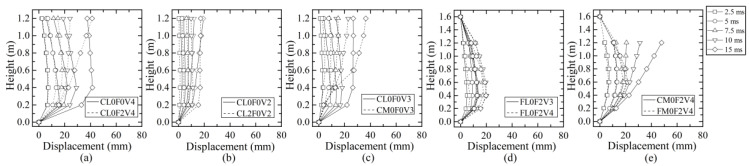
Parametric effect on lateral displacement distribution: (**a**) number of CFRP layers; (**b**) axial compression ratio; (**c**) impact height; (**d**) impact velocity; (**e**) boundary condition.

The lateral displacement distribution curves of some typical columns at some time points are compared in Figure 10. In the initial Stage-I before 2.5 ms, the lateral displacement of the column locally occurred near the impact region with a similar curve shape. At 2.5 ms after entering Stage-II, the lateral displacement was distributed along the whole height of the column and rendered different curve shapes for different cases. As shown in Figure 10a, the plain column CL0F0V4 developed an obvious shear-dominant distribution curve with large displacement at the impact region, while the displacement distribution curve was prone to be flexure-dominant for strengthened column CL0F2V4 with two-layer CFRP wraps because CFRP could enhance the impact resistance, restrict the shear damage, reduce the displacement, and even change the response mode from shear to flexure.

Figure 10b shows the effect of axial compression ratio *μ* on displacement distribution curves. With the increase in *μ* from 0 to 0.2, column CL2F0V2 presented an almost similar shear-dominant distribution curve to column CL0F0V2, despite the slightly large lateral displacement. This was because column CL0F0V2 tended to suffer shear failure under impact and the application of axial force would increase the lateral displacement. From Figure 10c, the variation in impact height *h* on the column would produce a different effect. When *h* increased from 400 mm to 600 mm, the shear-dominant distribution curve of column CL0F0V3 changed to be flexure-dominant for CM0F0V3, which was due to the decrease in flexural resistance. By making a comparison among columns CL0F0V2, CL0F0V3, and CL0F0V4, it was found that the displacement distribution almost rendered a similar curve shape with the increase in impact velocity but had different magnitudes. The effect of impact velocity *v* on displacement distribution curves is presented in Figure 10d. The increase in impact velocity resulted in an almost similar curve shape for fixed-hinged columns, but obviously increased the displacement magnitude. Figure 10e gives the comparison of displacement distribution curves between the cantilever column and fixed-hinged column. Due to the effect of the boundary condition at the top column, the distribution curve from the impact height to the top was different. The cantilever column had an almost linear increase, while the fixed-hinged column gradually decreased to zero in a bending deformation pattern. Below the impact height, both types of columns showed similar distribution curve shapes, but the fixed-hinged column developed a smaller displacement because of the enhanced lateral stiffness provided by the top support.

## 5. Discussion

### 5.1. Maximum Dynamic Response

**Figure 11:**
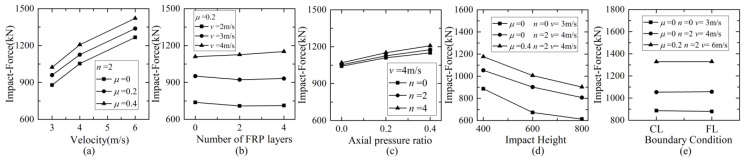
Maximum impact force versus: (**a**) impact velocity; (**b**) number of CFRP layers; (**c**) axial compression ratio; (**d**) impact height; (**e**) boundary condition.

The relationship curves between maximum impact force and different parameters are shown in Figure 11. The maximum impact force increased greatly with the increase in impact velocity regarding other parameters. However, once the impact velocity reached a certain value, the growth rate slowed down, which might be because the column would be subjected to serious damage and be unable to resist the impact with higher velocity. The increase in the layer number of CFRP wraps from zero to four had little influence on the maximum impact force. The reason might be that the contact stiffness of the impact region was enhanced less by CFRP wraps compared to pure concrete. With the increase in the axial pressure ratio from 0 to 0.4, the maximum impact force increased by about 20%. When the impact height increased from 400 mm to 800 mm, the maximum impact force tended to decrease. The maximum impact force was almost independent of the boundary condition at the top of the column regardless of the cantilever and hinge, which showed similar values. Hence, the maximum impact force was more sensitive to impact velocity, axial compression ratio, and impact height, and less sensitive to the CFRP layer and the top boundary condition.

**Figure 12:**
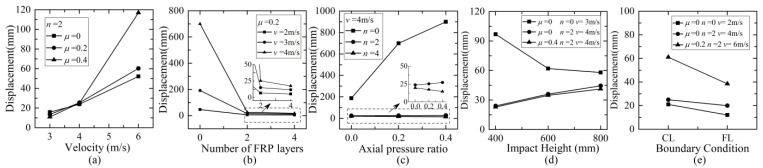
Maximum lateral displacement at the impact height versus: (**a**) impact velocity; (**b**) number of CFRP layers; (**c**) axial compression ratio; (**d**) impact height; (**e**) boundary condition.

Figure 12 gives the relationship curves between maximum lateral displacement at the impact height and different parameters. Note that if the case suffered severe damage, the maximum lateral displacement at the impact height would be determined as the value corresponding to the time moment of 80 ms after impact. The maximum lateral displacement increased almost in an exponential pattern with the increase in impact velocity. When the number of CFRP layers increased from 0 to 2, the maximum lateral displacement decreased significantly, especially under the impact at higher velocity, and just slightly when increasing to 4 layers. This was because two layers of CFRP wraps could sufficiently improve the impact resistance to reduce the damage and lateral deformation, and extra CFRP wraps had a similar effect under the same impact velocity. With the increase in the axial pressure ratio, the maximum lateral displacement increased greatly for plain columns and slightly for strengthened columns, and even decreased for columns with more CFRP. This might be because the plain columns were prone to suffer different degrees of shear damage, and the existence of the axial force would aggravate the axial movement along the inclined shear crack and result in the increase in lateral displacement. Strengthening with CFRP wraps would enhance the lateral impact resistance and also the axial load capacity of the column, and accordingly restrict the shear damage. With the increase in impact height, the maximum lateral displacement of plain columns decreased significantly due to the change from shear failure to the flexure-dominant mode, while strengthened columns presented a slight increase, because the influence of the flexure response mode was more significant. When the boundary condition at the column top changed from cantilevered to hinged, the maximum lateral displacement decreased due to the enhancement of lateral stiffness provided by the horizontal support at the top of the column.

### 5.2. Shear Force and Flexural Moment

**Figure 13:**
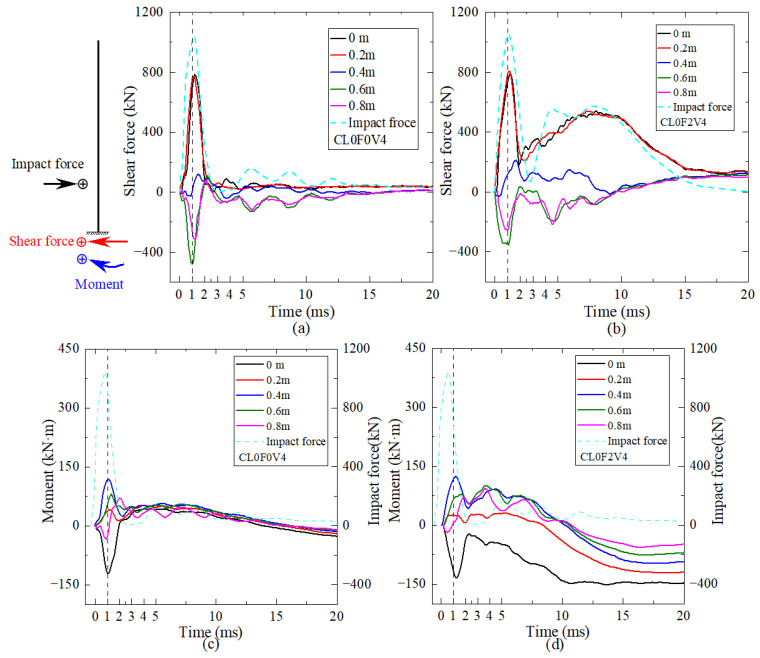
Time history curves: (**a**) shear force of column CL0F0V4; (**b**) shear force of column CL0F2V4; (**c**) bending moment of column CL0F0V4; (**d**) bending moment of column CL0F2V4.

Figure 13 shows the time histories of shear force and flexural moment at the different height of columns CL0F0V4 and CL0F2V4 under the same impact scenarios. Note that the positive direction of shear force and flexural moment is schematically drawn. In the initial 2 ms stage when the impact force underwent the triangular main pulse, all the time histories of shear force and bending moment at different heights showed similar triangular curves but had different activation times, which was induced by the transmission of a stress wave along the column height. The closer to the impact height, the earlier the activation time. It was observed that the column part above the impact height of 0.4 m developed a negative shear force and positive moment, which was opposite to that below the impact height. This was due to the large inertial force at the upper part after impact.

**Figure 14:**
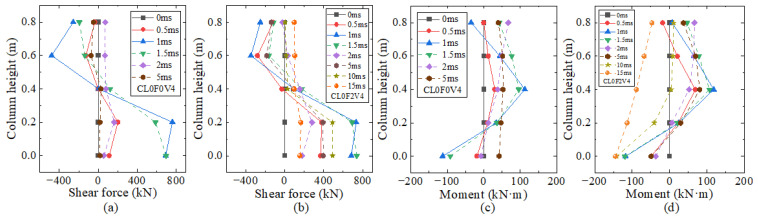
Distribution along the column height: (**a**) shear force of column CL0F0V4; (**b**) shear force of column CL0F2V4; (**c**) bending moment of column CL0F0V4; (**d**) bending moment of column CL0F2V4.

After the peak, the shear force of plain column CL0F0V4 decreased rapidly to zero due to severe shear failure, while the moment underwent a gradual decrease to zero and maintained an identical direction after about 2 ms, which might be due to the contribution of column longitudinal rebars after shear failure. For column CL0F2V4 wrapped with the two-layer CFRP, the shear force and moment curves obtained an increase again to the second peak and then gradually decreased to zero. The reason might be that strengthening with two-layer CFRP wraps improved the impact resistance of column CL0F2V4 to prevent the shear failure that occurred in column CL0F0V4, and ensured enough residual resistance to respond in a flexure-shear mode. The moment at different heights changed to the identical direction after about 10 ms, which was possibly due to the reduction in inertial force. The lateral distribution curves of shear force and flexural moment along the height at different time points for cantilever columns CL0F0V4 and CL0F2V4 are shown in Figure 14. It can be seen that the distribution curves of shear force and moment were different from that of the cantilever column under static horizontal concentrated load. Before 2 ms, the shear force was almost distributed anti-symmetrically with the impact height of 0.4 m, but the lower part developed a larger shear force. The moment distribution curve implied that there was an action of horizontal constraint near the column height of 0.8 m, which was explained by the phenomenon of impact effective length. Compared with column CL0F0V4, column CL0F2V4 developed an almost similar maximum shear force and moment at the lower part, but had a resistance improvement from CFRP wraps to avoid the shear failure and responded in the flexure mode, evidenced from the moment distribution curve at 15 ms.

### 5.3. CFRP Stress in Fiber Direction

**Figure 15:**
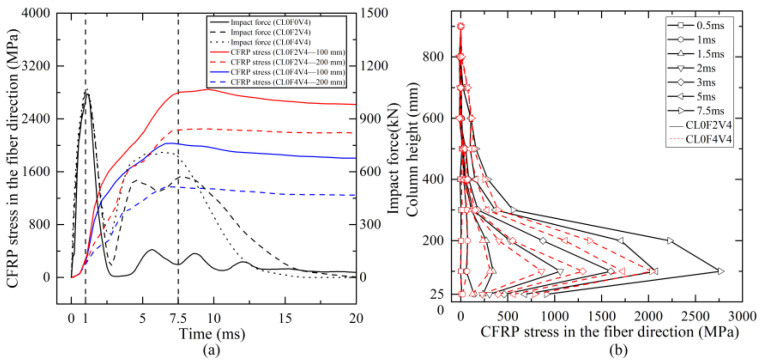
Response curves of CFRP stress: (**a**) time histories at different heights; (**b**) distribution along the column height.

Figure 15a shows the time histories of average stress of a CFRP hoop in the fiber direction at the column heights of 100 mm and 200 mm for cantilever columns CL0F2V4 and CL0F4V4 under the same impact. Note that the average stress was determined by averaging the stress of all the CFRP elements at the same column height. From Figure 15a, it can be observed that before 1 ms corresponding to the peak impact force, the average stress of CFRP at the heights of 100 mm and 200 mm was both small and close, regardless of different CFRP layers, indicating that the shear failure of the column did not occur at the stage of peak impact force, because CFRP did not yet fully work and the RC column mainly sustained the impact shear force by itself at this stage.

Thereafter, the CFRP stress increased rapidly along with the decrease and increase in impact force. During the stage when the impact force increased to the second lower peak, the CFRP stress almost reached the peak, indicating full engagement. The response of the strengthened column at this stage was quite different from that of the plain column, which was subjected to shear failure. This implied that the shear failure of the column under lateral impact occurred at the second increase stage of the impact force, and strengthening with CFRP wraps could prevent the shear failure mainly at this stage. After the peak, the CFRP stress underwent a small decrease and then remained stable with high residual stress. The stress level of CFRP wraps at the 100 mm height was larger than that at the 200 mm height. In addition, column CL0F4V4 having four-layer CFRP wraps developed lower stress than column CL0F2V4 with two layers. This might be because more CFRP wraps could enhance the resistance and reduce the damage, thus leading to a lower development of fiber stress. Figure 15b gives the lateral distribution of average stress of CFRP wraps within the column height of 900 mm for columns CL0F2V4 and CL0F4V4. It was seen that the stress was small along the column height at the time of 1.0 ms corresponding to the peak impact force, further proving the non-engagement of CFRP wraps at the initial stage. The CFRP wraps below the impact height developed a larger stress than that above. From the impact height to the bottom, the CFRP stress tended to increase, due to the shear damage close to the bottom of column. This indicated that the shear damage of column concrete would have influence on the engagement of external CFRP wraps.

## 6. Conclusions

This paper established FE models of reinforced concrete columns with or without CFRP wrapping from the literature, and FE models were then reasonably verified by a detailed comparative analysis between numerical results with experimental results. Based on the high-fidelity FE models, parametric analyses were then performed to investigate the effect of parameters on the dynamic response such as the boundary condition at the column top, layer number of CFRP wraps, axial compression ratio, impact velocity, and impact height. The following conclusions can be drawn based on numerical results.

(1) The dynamic response of columns with or without CFRP wrapping was more sensitive to impact velocity than other parameters, including impact force, lateral displacement, and damage pattern. With the increase in impact velocity, the damage of columns would become more severe, and the first peak of impact force and lateral displacement would increase rapidly.
(2) Increasing the layer number of CFRP wraps could improve the impact resistance of RC columns to sustain the impact with higher critical velocity, reduce lateral displacement, and effectively change the shear damage mode to the flexural damage mode. More CFRP wraps had almost no influence on the first peak of impact force, but increased the second peak.
(3) The effect of axial compression ratio on column damage would depend on the critical velocity of impact. If the impact with lower velocity was insufficient to cause shear damage, the application of axial compression ratio would be beneficial to enhance the impact resistance of the column and reduce the damage, and vice versa.
(4) The increase in impact height would change the response mode of plain RC columns but have little influence on that of strengthened columns. The variation in boundary condition at the column top from the cantilever to hinge had little influence on the damage mode of columns and the first peak of impact force, but it could reduce the lateral displacement due to the improvement of lateral stiffness.

## Figures and Tables

**Table 1:** Parameters of RC column specimens [21,23,33].

Specimen Group	Specimen No.	Longitudinal Rebars (mm)	Hoops (mm)	Cylinder Concrete Strength (MPa)	Layer Number of CFRP Wraps
C2	C2H0	16 ϕ 8	ϕ 6.5@100	28.0	0
	C2H2	16 ϕ 8	ϕ 6.5@100	30.5	2
C3	C3H0	16 ϕ 12	ϕ 6.5@330	29.6	0
	C3H4	16 ϕ 12	ϕ 6.5@330	32.2	4
F2	F2H0	16 ϕ 8	ϕ 6.5@100	29.8	0
	F2H2	16 ϕ 8	ϕ 6.5@100	29.8	2
F3	F3H0	16 ϕ 12	ϕ 6.5@330	29.6	0
	F3H4	16 ϕ 12	ϕ 6.5@330	32.2	4

**Table 2:** Material parameters of steel rebars [21,23,33].

Rebar Types	Diameter (mm)	Yielding Strength (MPa)	Ultimate Tensile Strength (MPa)	Modulus of Elasticity (MPa)
ϕ 6.5	6.5	427	483	204
ϕ 8	8	436	632	175
ϕ 12	12	471	610	194

**Table 3:** Parametric values.

Parameters	Values
Boundary condition at both ends	cantilever (C-series), fixed-hinged (F-series)
Axial compression ratio (*μ*)	0, 0.1, 0.2, 0.4
Layer number of CFRP wraps (*n*)	0, 2, 4
Horizontal impact height (mm) (*h*)	400 (L), 600 (M), 800 (H)
Impact velocity (m/s) (*v*)	2, 3, 4, 6, 8, 10

## Data Availability

Not applicable.
